# Transcriptomes of human prostate cells

**DOI:** 10.1186/1471-2164-7-92

**Published:** 2006-04-25

**Authors:** Asa J Oudes, Dave S Campbell, Carrie M Sorensen, Laura S Walashek, Lawrence D True, Alvin Y Liu

**Affiliations:** 1 Urology, University of Washington, 1959 NE Pacific St., Seattle, WA 98195-6510, USA; 2 Pathology, University of Washington, 1959 NE Pacific St., Seattle, WA 98195-6100, USA; 3 Institute for Systems Biology, 1441 N 34^th ^St., Seattle, WA 98103, USA

## Abstract

**Background:**

The gene expression profiles of most human tissues have been studied by determining the transcriptome of whole tissue homogenates. Due to the solid composition of tissues it is difficult to study the transcriptomes of individual cell types that compose a tissue. To overcome the problem of heterogeneity we have developed a method to isolate individual cell types from whole tissue that are a source of RNA suitable for transcriptome profiling.

**Results:**

Using monoclonal antibodies specific for basal (integrin β4), luminal secretory (dipeptidyl peptidase IV), stromal fibromuscular (integrin α 1), and endothelial (PECAM-1) cells, respectively, we separated the cell types of the prostate with magnetic cell sorting (MACS). Gene expression of MACS-sorted cell populations was assessed with Affymetrix GeneChips. Analysis of the data provided insight into gene expression patterns at the level of individual cell populations in the prostate.

**Conclusion:**

In this study, we have determined the transcriptome profile of a solid tissue at the level of individual cell types. Our data will be useful for studying prostate development and cancer progression in the context of single cell populations within the organ.

## Background

Prostate cancer is the second leading cause of cancer death among American men [1,2]. Due to the high incidence of prostate cancer the biology of the organ has been extensively studied. Crucial to our understanding of the cancer process is the biology of prostate development, in particular, the gene expression changes that accompany epithelial cell differentiation. DNA microarray technology has revolutionized the field of gene expression profiling and has seen wide application in the study of prostate cancer [3-5] as well as other types of cancer [6]. A byproduct of these studies is the gene expression profiles of most tissues of the human body have been assessed (e.g., the Novartis GeneAtlas). The next step in understanding how a tissue functions at a molecular level is to determine the transcriptome profile of individual cell types that constitute the tissue. Cell-type specific transcriptomes will allow us to more precisely define prostate cell lineages, cell-cell interactions, autocrine or paracrine signaling pathways, and would be useful to identify biomarkers for diseases such as cancer.

The main problem encountered in studies of cells from solid tissue has always been determining a method to separate the cells of interest from the organ. Our solution to the cell separation problem was to use collagenase digestion of tissue combined with cell sorting based on cell-type specific cluster designation (CD) antigens [7]. A comprehensive CD phenotyping of the constituent cell types of the prostate [8] now makes it possible to sort most cell populations from the tissue by flow cytometry. The use of antibodies targeting cell-surface CD markers to separate blood cells by fluorescence-activated cell sorting (FACS) is well established, and has been used for microarray analysis [9-11]. In this study, we used magnetic cell sorting (MACS) to isolate the major prostatic cell types: luminal secretory and basal cells of the epithelium, and fibromuscular cells of the stroma for transcriptome profiling. Endothelial cells were also sorted to represent a cell type not specific to the prostate. Analysis of the transcriptomes identified cell specific gene expression signatures and paracrine signaling pathways that are potentially active within the prostate.

## Results

### Specificity of antibodies used in MACS

**Figure 1:**
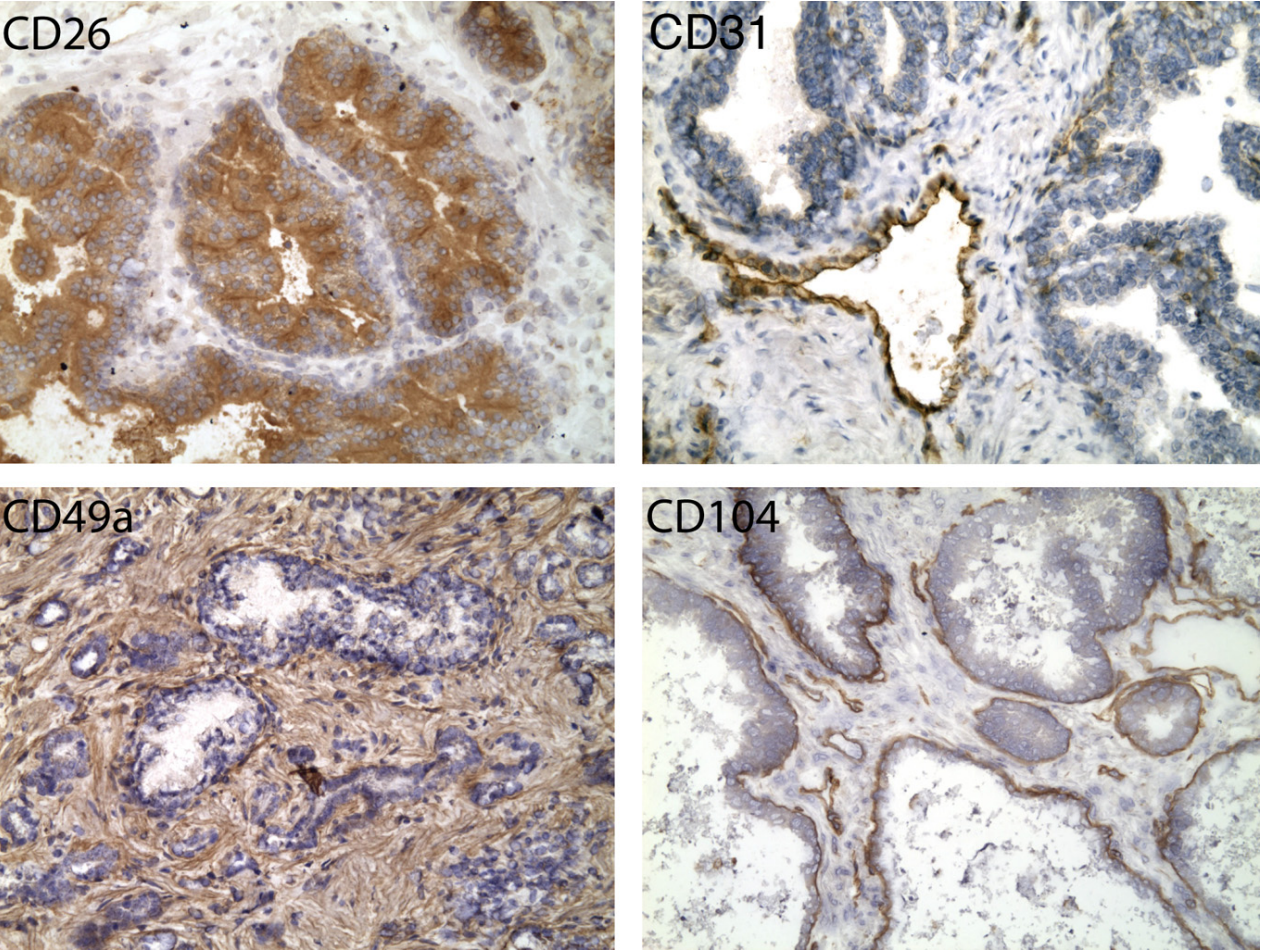
**Cell type specific CD antibodies. **The specificity of antibodies directed at luminal secretory (CD26), endothelial (CD31), stromal fibromuscular (CD49a), and basal epithelial (CD104) cells are demonstrated. Brown color indicates positive staining. Each prostatic gland is composed of a one-cell layer thick basal epithelium surmounted by columnar secretory cells enclosing the luminal space. The interglandular stroma contains the fibromuscular cells and various other cell types.

The CD molecules targeted by MACS were CD26 (dipeptidyl peptidase IV) for luminal cells, CD104 (integrin β4) for basal cells, CD49a (integrin α 1) for stromal fibromuscular cells, and CD31 (PECAM-1) for endothelial cells. Immunohistochemistry performed with the antibodies that were used for MACS sorting is shown in Fig. 1 and confirms their specificity for the cell-types that were targeted. The antibodies that primarily recognize basal epithelial cells and luminal secretory cells (CD104 and CD26 respectively) show some staining of endothelial cells [8]. Therefore, the endothelial cell data was used to filter out genes expressed by endothelial cells from the CD104 and CD26 transcriptomes.

### Cell type-specific transcriptomes

**Figure 2:**
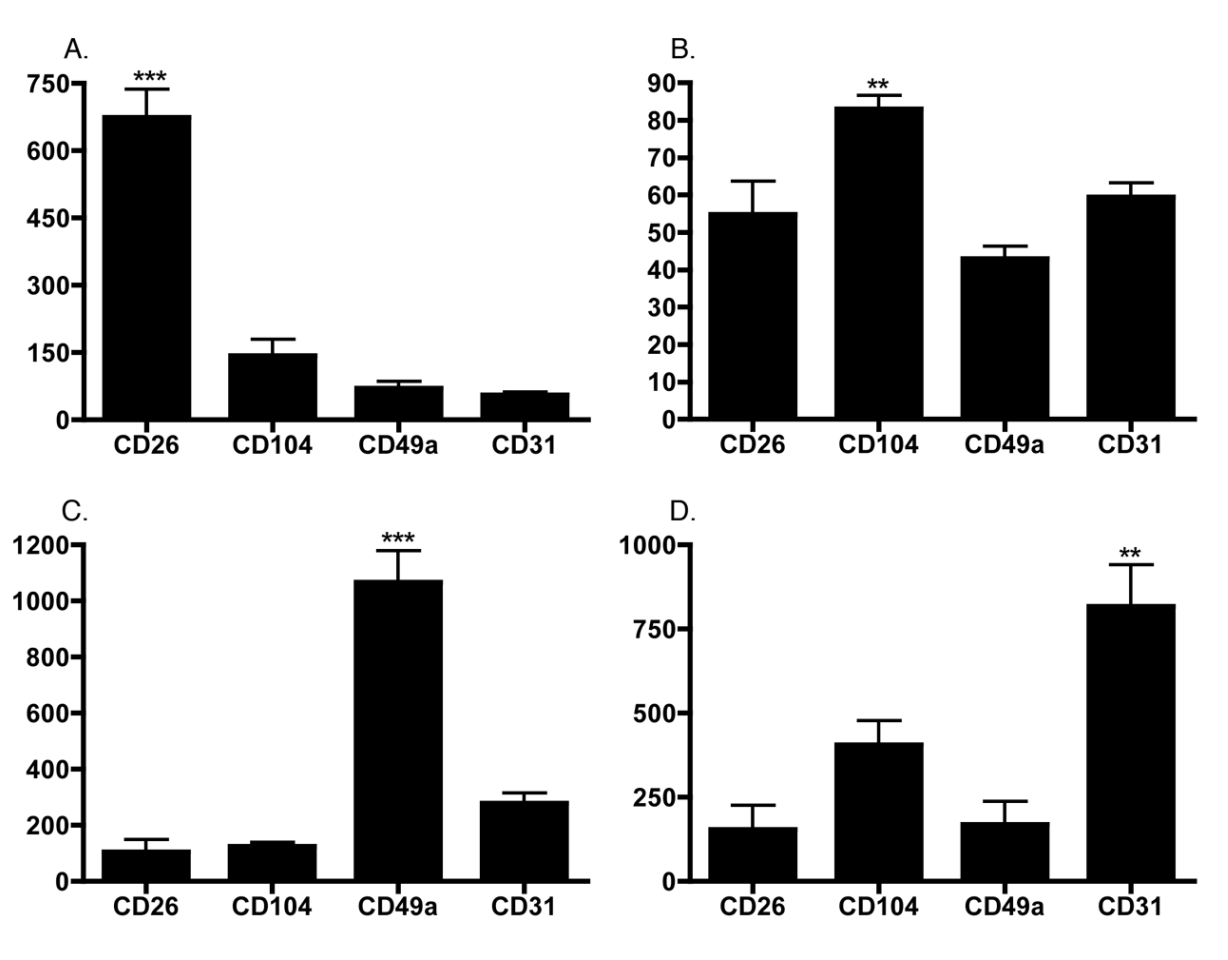
**Effectiveness of cell sorting. **Differential expression of transcripts that code for surface antigens targeted by MACS sorting. (A) luminal (CD26), (B) basal (CD104), (C) stromal (CD49a), and (D) endothelial (CD31). ANOVA results indicate significantly different expression at P < 0.001 (***) and P < 0.01 (**). Cell sorting enriched for the transcripts belonging to the gene that codes for the surface antigen that was the target of cell sorting.

**Figure 3:**
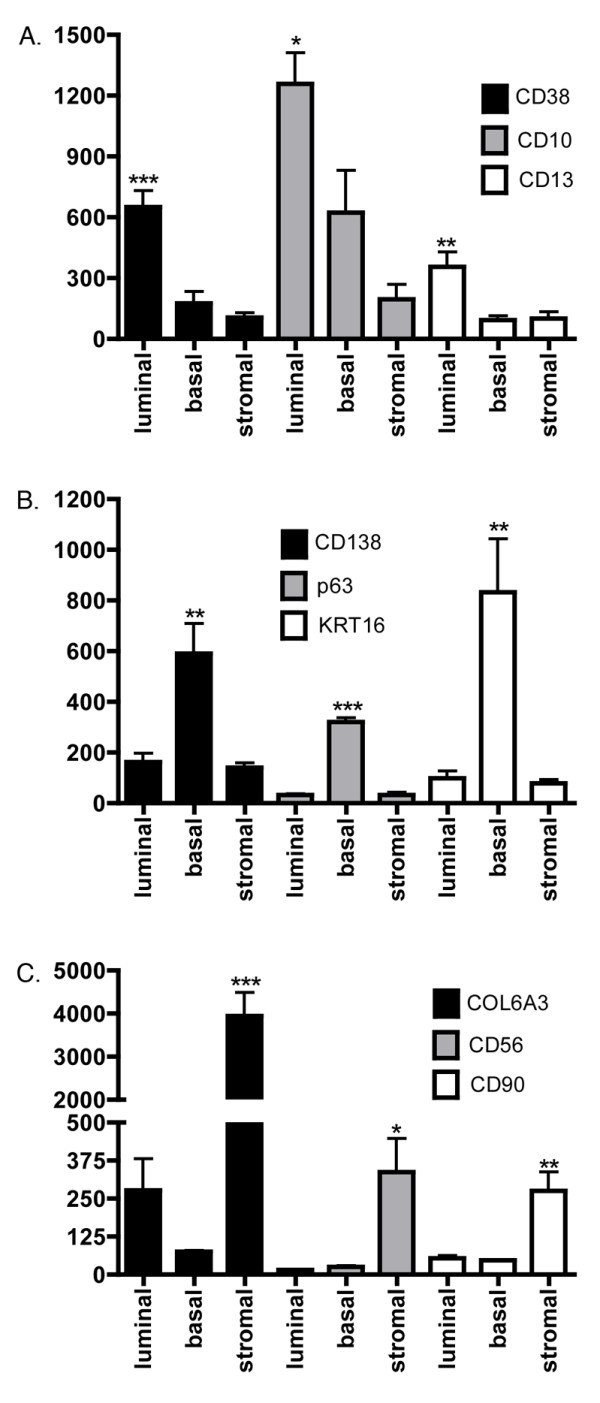
**Prostate cell specific genes. **Expression levels of genes that are documented in the literature as being expressed by prostate luminal secretory (A), basal epithelial (B), or stromal fibromuscular (C) cells was determined from Affymetrix GeneChip data. ANOVA results indicate significantly different expression at P < 0.001 (***), P < 0.01 (**), and P < 0.05 (*).

**Figure 4:**
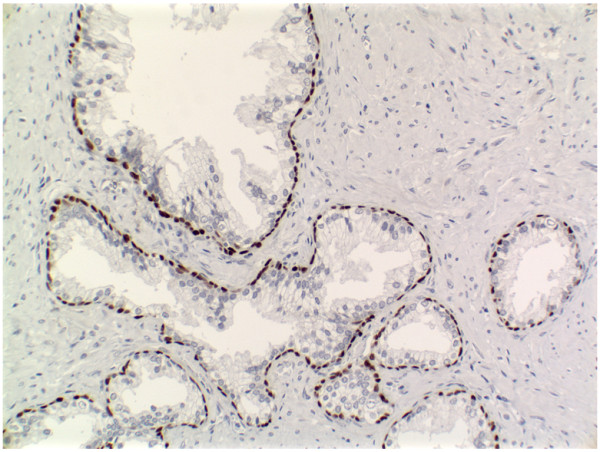
**p63 Immunohistochemistry. **Immunohistochemistry detecting p63 in the prostate. Brown color indicates positive staining. The expression of p63 protein in the prostate is confined to basal cells, which corresponds to the expression of its transcript in our data.

Microarray expression data from the four prostate cell types were compared with each other. Raw data is available in the NCBI GEO database under the accession number GSE3998, from our website [12], and in Softmatrix format (see Additional file 1). ANOVA was performed on the data (5 biological replicates per cell type) to determine the effectiveness of MACS at enriching the signal of transcript from the target antigen (Fig. 2). The CD26 and CD49a transcripts were differentially expressed in their respective sorts relative to the other sorts (P < 0.001), as were CD31 and CD104 transcripts (P < 0.01), thereby confirming the effectiveness of our sorts. To further assess whether the populations of sorted cells provide accurate representation of the targeted prostate cell types, we compared the expression of genes documented in the literature as primarily expressed by luminal, basal, or stromal cells with our sorted luminal, basal, and stromal datasets (Fig. 3). The luminal cell genes CD10, CD13 and CD38 [13-15] were all found to be differentially expressed in the CD26 populations (P < 0.05). The basal cell genes CD138, p63, and KRT16 [8,16,17] were also differentially expressed in the CD104 populations (P < 0.01). Finally, the stromal cell genes COL6A3, CD56, and CD90 [8,18,19] were differentially expressed in the CD49a populations (P < 0.05). Many of the documented cell type-specific genes such as p63 were detected at or near background intensity level in cells that were not targeted by sorting. The specificity of p63 protein expression to basal cells was verified by immunohistochemistry and confirmed the array data (Fig. 4). These analyses indicated that the transcriptomes generated by MACS of prostate tissue were representative of luminal, basal and stromal cells.

Comparison of cell type transcriptomes with whole tissue

To assess the value of cell sorting in transcriptome profiling we compared sorted cell expression profiles with those of whole prostate tissue archived in the Novartis GeneAtlas project [6]. The data from GeneAtlas was produced with custom Affymetrix human GeneChips. The GeneChips we used had 22,215 probesets in common with the arrays used in the GeneAtlas project. The 22,215 probesets represent 15,049 genes. Comparison of the transcriptomes revealed that 3,419 genes were detected in our sorted cell transcriptomes but not in the whole tissue (see Additional file 2). The detection of genes in sorted cells but not whole tissue indicated an enrichment of cell-type specific genes by sorting. Conversely, a list of genes detected in whole tissue but not in sorted cells was also compiled. Initially, there were 1,828 genes in the list, detection of which could be attributed to blood cells and other non-targeted cell types present in whole prostate tissue. To account for the genes that appeared in whole tissue but not sorted cells we subtracted GeneAtlas data for whole blood *in silico*, which reduced the number of genes by 80% to 352. Subtracting datasets for CD8^+^, CD4^+^, CD34^+^, and CD14^+ ^cells, which were shown in our previous work [8] to be present in the prostate, reduced the number of genes only detected in whole tissue to 233 (see Additional file 3). Of the remaining genes only 14 were detected at signal levels greater than 100, which was considered to represent moderate expression (see Additional file 4). Overall the sorted cell dataset included 98.5% of the genes detected in the whole prostate plus an additional 3,419 genes not detected in whole tissue.

### Prostate luminal, basal, and stromal genes

**Figure 5:**
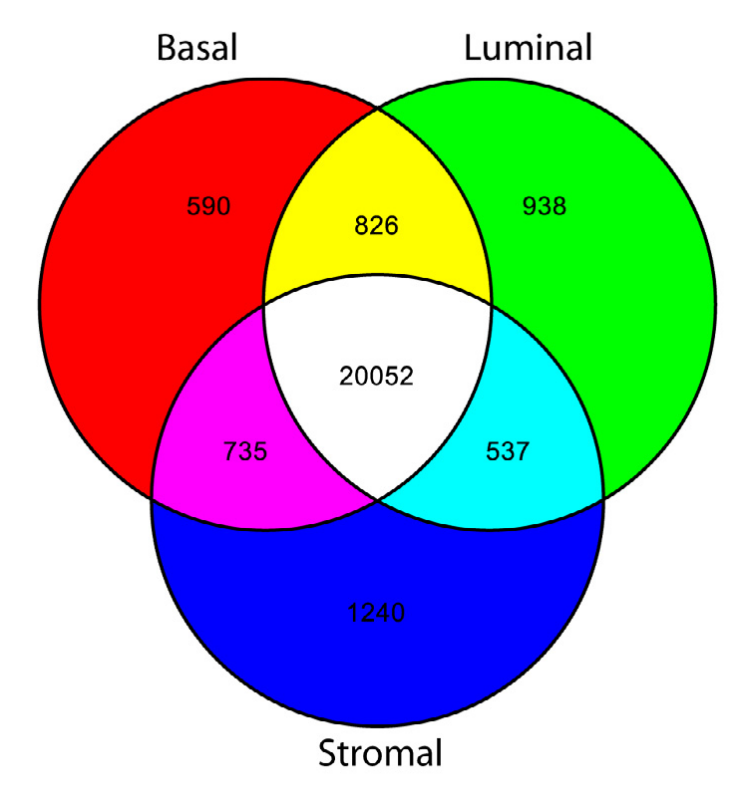
**Similarity of prostate cell transcriptomes. **A Venn diagram depicting the number of genes expressed by each prostate cell type: basal (red), luminal (green), and stromal (blue). Intermediate colors represent groups of genes that are shared between two cell types. Genes expressed by all prostate cell types are white. The transcriptomes of luminal cells are more similar to basal cells than stromal cells.

Our next analysis was aimed at determining which genes were uniquely detected in each cell type and could therefore serve as an expression signature for that cell type. Comparison of the genes detected in each sort by Venn diagram revealed an expected similarity between the epithelial cell types where luminal and basal cells have roughly twice the number of genes in common than do luminal and stromal cells (Fig. 5). To identify significant unique expression, ANOVA was performed for the genes determined to be present in the sorted cells. A total of 197, 150, and 632 genes had significant P-values of < 0.05 in luminal, basal, and stromal sorts respectively (see additional files 5, 6, 7). These genes are likely to represent a cell specific signature for the corresponding cell types. We also note that for some highly expressed genes such as PSA signal is detected in non-specific cell types (basal and stromal in the case of PSA). The non-specific detection is due to low levels of contaminating cells in a sort (for example luminal cells in the basal sort), however, it is clear from statistical analysis that the only cell type where PSA shows significant expression is the luminal cells, as would be expected.

### Cell specific signaling pathways

**Figure 6:**
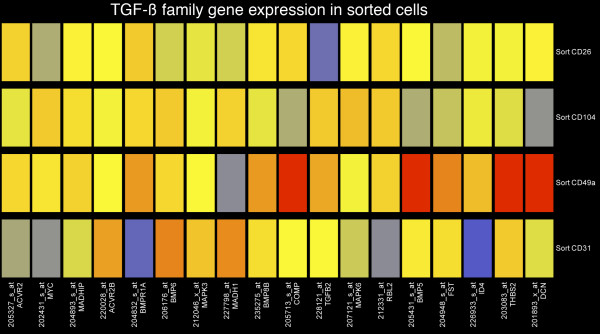
**Cell type specific expression of TGFβ family members. **A heatmap diagram of the expression profiles for genes that belong to the TGFβ and related signaling pathways. Red indicates high expression and blue indicates no detectable expression. Prostate stromal cells differentially express genes involved in TGFβ signaling relative to other prostate cell types.

**Figure 7:**
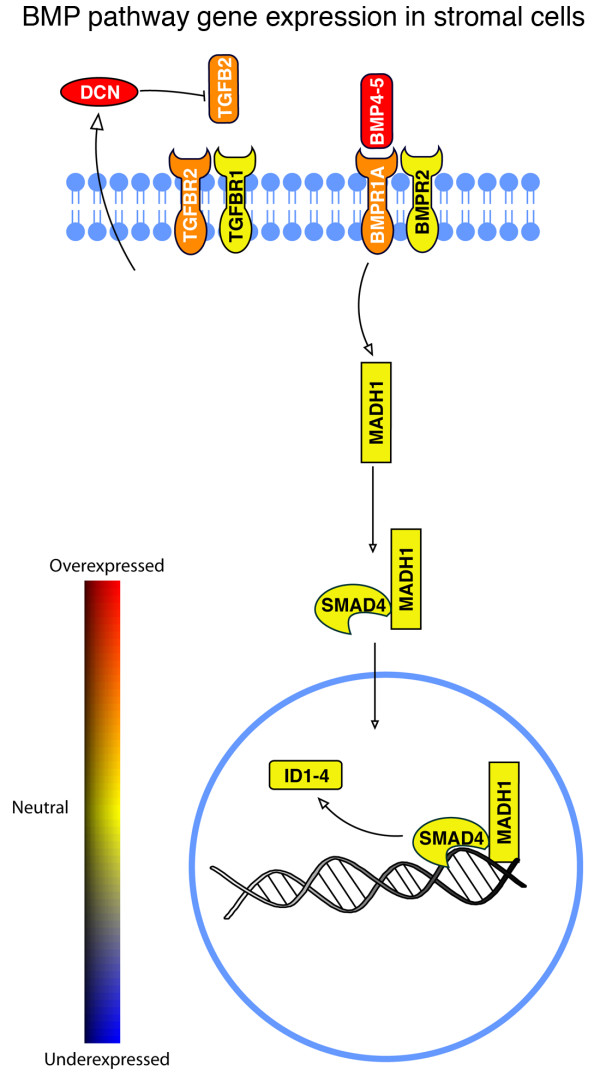
**Transcript expression of BMP signaling pathway members. **The diagram indicates cellular locations of differentially expressed genes in the BMP signaling pathway in prostate stromal cells. Decorin, a negative regulator of TGFβ signaling, is produced by stromal cells, which may result in autocrine regulation of the pathway. BMP4 and BMP5 are produced by stromal cells and may be responsible for paracrine regulation of Inhibitor of Differentiation (ID) gene expression in the prostate.

Determination of cell specific transcriptome profiles allowed us to examine the differential expression of genes involved with signal transduction in the prostate. We chose to investigate the expression of TGFβ family members by applying our expression signatures to the genes that compose the TGFβ pathway as compiled in the Kyoto Encyclopedia of Genes and Genomes (KEGG). A group of genes that are assigned to the KEGG TGFβ signaling pathway were significantly differentially expressed as determined by ANOVA (P < 0.05) in prostate stromal cells relative to basal, luminal, and endothelial cells (Fig. 6). The analysis suggested that in prostate stromal cells, autocrine signaling via the classic TGFβ pathway might be reduced by high expression of decorin. TGFβ paracrine signaling from stromal cells to basal and luminal cells appears to occur via TGFβ2 since the basal and luminal cells express TGFβ receptors but low to undetectable levels of TGFβ2. However, autocrine and paracrine signaling in the prostate via the bone morpohgeneic protein (BMP) signaling-pathway (Fig. 7), which is closely related to TGFβ and is a component of the KEGG TGFβ signaling system, could be enhanced by high expression of BMP4 and BMP5 by stromal cells.

## Discussion

Our approach to cell specific transcriptome profiling of solid tissues involves first identifying the specificity of CD cell-surface markers for cell types within the tissue. CD markers with the proper specificity can then be used to target cell types for isolation by cell sorting techniques such as MACS. We chose MACS as the separation method for this study because the procedure is much faster than FACS (minutes vs. hours) and allowed us to minimize the time cells were handled. We determined transcriptome profiles of four main cell types in the prostate: luminal secretory, basal epithelial, stromal fibromuscular, and endothelial cells. It is possible the approximately 18-hour time frame from surgical removal to cell sorting and storage contributed to some differences in the transcriptomes of luminal, basal, and stromal cells. Reduction of the processing time should be considered in future studies. However, when we examined MACS-sorted prostate cell transcriptomes, the effectiveness of our approach was demonstrated by the fact that luminal secretory, basal epithelial, and stromal fibromuscular transcriptomes differentially expressed genes that are well documented as specific to the prostate cell type we intended to capture by sorting. The transcriptomes revealed that luminal secretory cells were more similar to basal epithelial cells and less similar to stromal fibromuscluar cells, as would be expected for the two epithelial cell types.

The utility of cell specific transcriptomes is demonstrated by the identification of genes predominantly expressed in luminal, basal, or stromal fibromuscular cells of the prostate. To investigate paracrine signaling within the prostate we applied the expression data from our cell specific groups to the genes of the TGFβ and related signaling pathways (as compiled in KEGG), which allowed us to identify which cells were expressing components of the pathways in the prostate. We chose to investigate the TGFβ pathway because it had been extensively studied in the prostate and is known to be involved in control of apoptosis and growth of the prostate epithelium [20]. Our data showed that in the cell types we assayed stromal fibromuscular cells differentially expressed TGFβ pathway members. Interestingly, the activity of classical TGFβ signaling through TGFβ receptors may be reduced in prostate stromal fibromuscular cells through an autocrine mechanism mediated by the expression of a negative regulator of TGFβ signaling decorin [21]. Paracrine signaling from stromal fibromuscular to basal cells also is likely to occur in the prostate since TGFβ receptors I and III are expressed by basal cells.

Although our data indicates that TGFβ signaling via the classic pathway is reduced in stromal fibromuscular cells, activity of the closely related bone morphogenic protein (BMP) signaling pathway appears to be enhanced. One result of BMP signal transduction is production of inhibitor of differentiation (ID) proteins [22,23]. ID proteins are negative regulators of basic helix-loop-helix transcription factors and have been shown to be important in development, the cell cycle, and tumorigenesis. Our data indicates that luminal, basal, and stromal cells of the prostate express all four isoforms of the ID genes at high levels and that the expression of IDs may be controlled by BMP4 and BMP5 produced by stromal cells. BMP4 is known to inhibit ductal budding during development of the mouse prostate [24]. Recent work in a mouse model system has shown that BMP4 heterozygous adult animals have enlarged prostates due to increased branching of the prostate ducts during neonatal development [25]. BMP4 has also been shown to inhibit growth of the prostate cancer cell line LNCaP [26] and induce the expression of ID1, ID2, and ID3 in cell lines [23]. Little is know about BMP5 with regard to the prostate, although its expression has been observed to be increased in benign prostatic hyperplasia [27]. Our data suggests that BMP signaling from the stromal fibromuscular cells to epithelial cells may be necessary to maintain the differentiated state of the prostate by inducing expression of ID family members 1–4 in these cells.

Transcriptomes of prostate epithelial, stromal, and endothelial cells provide the basis for assessing the degree to which genes associated with prostate cancer are abnormally expressed. Furthermore, cell specific transcriptomes also allow abnormally expressed genes to be assigned to a cell type in the prostate. Genes over-expressed in prostate cancer include AMACR, HPN, RDH11, and TMPRSS2 [28-31]. Confirmation that the source of these gene products is the cancer cell population of these tissues has been provided by immunohistochemical localization studies. Our investigation found that in benign tissue very low levels of AMACR and HPN transcripts were expressed only by luminal secretory cells, and not by other parenchymal cells, while RDH11 and TMPRSS2 were highly expressed in luminal secretory cells, which provides an indication that luminal cells are the origin of some cancers.

With respect to endothelial cells, virtually no work has been done investigating differential expression of molecular mediators of angiogenesis. We anticipate that defining endothelial gene expression patterns will be important in understanding prostate carcinogenesis, since tumor angiogenesis is a generic problem of significance in tumor biology, and studies of other tumors such as colon cancer have found it significant [32]. Our study provides a normal endothelial transcriptome profile that can serve as a reference for such future studies of prostate tumor angiogenesis.

## Conclusion

The transcriptomes of prostate luminal, basal, and stromal fibromuscular cells we have generated are the first such data determined for a solid tissue and are of value for the study of prostate cancer. Future investigation of the transcriptomes of CD sorted prostate cancer cell types will allow us to establish more fully their lineage relationship to the normal epithelial cell types and to discover aberrant signaling pathway activation or repression present in cancer. In conclusion, we have reported transcriptome profiles of individual cell types collected from live solid tissue and established a cell sorting methodology that can be used to isolate cells from other tissues which are suitable for transcriptome profiling.

## Methods

### Tissue samples

Prostate tissue specimens were obtained from radical prostatectomy surgeries at the University of Washington Medical Center and processed as previously described [7]. Briefly, surgically resected prostates were inked and sectioned to localize the cancer foci. Benign tissue samples were excised from cancer-free areas of the prostates. For sorting, the samples were minced and digested with collagenase in RPMI-1640 media supplemented with 5% fetal bovine serum overnight (approximately 18 hours) at room temperature on a magnetic stirrer. The cell suspension was filtered with a 70-μm Falcon cell strainer, diluted with an equal volume of Hanks' balanced salt solution (HBSS), and aspirated with an 18-gauge needle. A discontinuous density gradient made with Percoll (Amersham Pharmacia, Piscataway, NJ) was used to partition the cells into a stromal fraction (strom, ρ = 1.035) and an epithelial fraction (epi, ρ = 1.07). The strom and epi preparations were filtered with 40-μm Falcon cell strainers before labeling for MACS. CD49a^+ ^cells were sorted from strom while CD26^+ ^and CD104^+ ^cells were sorted from epi. CD31^+ ^cells were sorted from unbanded cells.

For immunohistochemistry with antibodies used for MACS, 5-μm thick, acetone fixed, frozen sections were used as described [8]. Each antibody was tested against prostate tissue from three different donors. The primary antibodies were R-Phycoerythrin (PE)-conjugated monoclonal antibodies (BD-PharMingen, San Diego, CA): CD26 (clone M-A261), CD31 (clone WM-59), CD49a (clone SR74), and CD104 (clone 439-9B) used at a titer of 1:25, 1:25, 1:25, and 1:60, respectively. Immunoreactivity was visualized with DAB reagents (Vector Labs, Burlingame, CA). Immunohistochemistry to detect p63 was performed on sections of formalin-fixed paraffin embedded tissue microwaved 15 minutes in 0.01 M citrate buffer for antigen retrieval. The primary monoclonal antibody for p63 was clone 4A4 (Dakocytomation, Carpinteria, CA) and was used at a titer of 1:500. Immunoreactivity of p63 was visualized with ABC reagents (Vector Labs). Immunostained sections were imaged with an Olympus BX41 microscope (Olympus, Melville, NY) equipped with a MircoFire digital camera (Optronics, Goleta, CA). Composite images were constructed with Photoshop CS (Adobe Systems, San Jose, CA), and all source images are available at the SCGAP website.

### MACS cell isolation

For stromal cells, the strom cells were resuspended in 0.1% bovine serum albumin (BSA)-HBSS, and 5 μl CD49a-PE was added for 15 min at room temperature in the dark. The reaction was stopped by the addition of 1 ml 0.1% BSA-HBSS and centrifugation. The labeled cells were resuspended and 15 μl paramagnetic microbead conjugated anti-PE antibody (Miltenyi Biotec, Auburn, CA) was added for 15 min. After incubation the positive and negative cells were separated in an AutoMACS cell sorter (Miltenyi Biotec, Auburn, CA) using a double positive sort program. Aliquots of positive and negative cell fractions were analyzed by FACS (Becton Dickinson, Mountainview, CA) to determine the purity of MACS samples; only >85% pure fractions were used for microarray experiments. The purity level was chosen due to our own observations (unpublished data) and studies by Szaniszlo and colleagues that showed a transcriptome of a 75% pure sorted cell population is largely identical to a 100% pure population [11]. The CD49a-positive cells were pelleted by centrifugation and resuspended in RLT lysis buffer (Ambion, Austin, TX) for storage at -80C. For epithelial cells, the epi cells were selected with either CD26-PE or CD104-PE. For endothelial cells, CD31-PE was used.

### Affymetrix expression profiling

MACS-sorted prostate cell lysates in RLT buffer were stored at -80C for no more than a month. Total cellular RNA was prepared with an RNaqueous kit (Ambion, Austin, TX). Quality and concentration of RNA was determined by using an Agilent 2100 Bioanalyzer with a RNA Nano Labchip assay (Agilent Technologies, Palo Alto, CA). Only RNA samples that were of sufficient concentration and showed no degradation were used for array hybridization.

Gene expression by sorted cells was analyzed with Human Genome U133 Plus 2.0 GeneChips (Affymetrix, Santa Clara, CA). Five separate biological replicates of each sorted cell population were assayed to produce a data set of 20 chips. The GeneChips were prepared, hybridized, and scanned according to the protocols provided by Affymetrix. Briefly, 200 ng of total RNA was reverse transcribed with a poly(T) primer containing a T7 promoter and the cDNA was made double-stranded. An in vitro transcription was performed to produce unlabeled cRNA. Next, 1st strand cDNA was produced from a random primed reaction. cDNA was made double stranded in a reaction with a poly(T) primer containing a T7 promoter. Finally, an in vitro transcription was performed with biotinylated ribonucleotides to produce biotin labeled cRNA. Labeled cRNA was then hybridized with the GeneChips. The chips were washed and stained with streptavidin-PE using an Affymetrix FS-450 fluidics station. Data was collected with an Affymetrix GeneChip Scanner 3000.

### Data analysis

CEL files produced by GeneChip Operating Software (Affymetrix) were loaded into GeneSpring 7.2 (Agilent Technologies) via the robust multiple array average (RMA) preprocessor. The GeneSpring RMA preprocessor uses the same analysis algorithm for Affymetrix array data as the open source Bioconductor project [33]. GeneSpring's replicate error model was used for our analysis. Genes in the dataset with an average raw fluorescence signal <50 were considered to be undetected by the experiment. Statistical significance of differential expression between luminal, basal, stromal fibromuscular, and endothelial sorts was determined by a parametric 1-way ANOVA using the Benjamini and Hochberg false discovery algorithm with P < 0.05. Pathway analysis was performed with GeneSpring's built-in functions utilizing human KEGG pathways release 33.0 [34]. The probeset comparison of whole prostate tissue was accomplished by using HG-U133A Affymetrix gene chip data in the form of CEL files published in the Novartis GeneAtlas [6]. All of the 22,215 HG-U133A probesets were exactly reproduced on the HG-U133 2.0 Affymetrix GeneChip used in our studies, therefore, the data could be directly compared between whole prostate and sorted cells without probeset bias. The 22,215 corresponding probesets represented 15,049 genes. The U133A CEL files from GeneAtlas were RMA normalized and compared with the U133 Plus 2.0 data from sorted cells using GeneSpring 7.2.

## Abbreviations

CD: Cluster designation

MACS: Magnetic cell sorting

FACS: Fluorescence activated cell sorting

cDNA: Complementary DNA

HBSS: Hanks balanced salt solution

BSA: Bovine serum albumin

cRNA: Complementary RNA

ANOVA: Analysis of variance

KEGG: Kyoto Encyclopedia of Genes and Genomes

TGFβ: Transforming growth factor beta

BMP: Bone morphogenic protein

ID: Inhibitor of differentiation

RMA: Robust multiple array average

## Authors' contributions

AO co-designed the study, carried out Affymetrix experiments, analyzed data, and prepared the manuscript. DC compared whole prostate data with sorted cell data. CS and LW sorted prostate cells with MACS and FACS systems and performed immunohistochemistry experiments. LT co-designed the study and contributed to the preparation of the manuscript. AL co-designed the study, performed immunohistochemistry experiments, and contributed to preparation of the manuscript.

## Supplementary Material

Additional file 1 **Softmatrix raw data file of prostate cell transcriptomes **RMA normalized raw Affymetrix signal for MACS sorted prostate cells in the Softmatrix format. Column ID_REF indicates Affymetrix probeset, CD26_repX indicates luminal cell sorts, CD104_repX indicates basal cell sorts, CD49a_repX indicates stromal cell sorts, and CD31_repX indicates endothelial cell sorts, where X is the biological replicate (1-5). Click here for file

Additional file 2 **Genes detected in sorted cells but not whole tissue **Column titles indicate the following: probeset-Affymetrix probeset ID, gene_symbol-HUGO gene name, public_ID-genebank ID of sequence used to make the probeset.  Column titles CD104, CD49a, CD31, CD26, and Prostate indicate sample name.  Expression levels are indicated by fluorescent signal intensity. Click here for file

Additional file 3 **Subtraction of blood-cell genes from prostate transcriptomes **Number of genes (y-axis) detected in whole prostate tissue but not sorted (x-axis point “Prostate”).  The remaining x-axis points indicate the blood-cell type which was subtracted *in silico* from the whole prostate data and the resulting number of genes is plotted on the y-axis.  Subtraction of whole blood expressed genes from whole prostate data accounted for 88% of genes detected in whole prostate but not sorted cells. Click here for file

Additional file 4 **Genes detected in whole prostate but not sorted cells **Column titles indicate the following: probeset-Affymetrix probeset ID, gene_symbol-HUGO gene name, public_ID-genebank ID of sequence used to make the probeset.  Column titles CD104, CD49a, CD31, CD26, Prostate, CD4, CD8, CD14, CD34, and wholeblood indicate sample name.  Expression levels are indicated by fluorescent signal intensity. Click here for file

Additional file 5 **Genes detected only in prostate luminal cells **Column titles indicate the following: probeset-Affymetrix probeset ID, X_raw-Affymetrix signal intensity, X_stdev-standard deviation of replicate samples where X=sorted cell sample name.  ANOVA P<0.05.  Expression levels are indicated by fluorescent signal intensity. Click here for file

Additional file 6 **Genes detected only in prostate basal cells ** Column titles are the same as additional file 5. ANOVA P<0.05 Click here for file

Additional file 7 **Genes detected only in prostate stromal cells **Column titles are the same as additional file 5. ANOVA P < 0.05. Click here for file

## References

[B1] Jemal A, Clegg LX, Ward E, Ries LA, Wu X, Jamison PM, Wingo PA, Howe HL, Anderson RN, Edwards BK (2004). Annual report to the nation on the status of cancer, 1975–2001, with a special feature regarding survival. Cancer.

[B2] Parker SL, Tong T, Bolden S, Wingo PA (1996). Cancer statistics, 1996. CA Cancer J Clin.

[B3] Foley R, Hollywood D, Lawler M (2004). Molecular pathology of prostate cancer: the key to identifying new biomarkers of disease. Endocr Relat Cancer.

[B4] Huppi K, Chandramouli GV (2004). Molecular profiling of prostate cancer. Curr Urol Rep.

[B5] Nelson PS (2004). Predicting prostate cancer behavior using transcript profiles. J Urol.

[B6] Su AI, Wiltshire T, Batalov S, Lapp H, Ching KA, Block D, Zhang J, Soden R, Hayakawa M, Kreiman G, Cooke MP, Walker JR, Hogenesch JB (2004). A gene atlas of the mouse and human protein-encoding transcriptomes. Proc Natl Acad Sci U S A.

[B7] Liu AY, True LD, LaTray L, Nelson PS, Ellis WJ, Vessella RL, Lange PH, Hood L, van den Engh G (1997). Cell-cell interaction in prostate gene regulation and cytodifferentiation. Proc Natl Acad Sci U S A.

[B8] Liu AY, True LD (2002). Characterization of prostate cell types by CD cell surface molecules. Am J Pathol.

[B9] Shim MH, Hoover A, Blake N, Drachman JG, Reems JA (2004). Gene expression profile of primary human CD34+CD38lo cells differentiating along the megakaryocyte lineage. Exp Hematol.

[B10] Zeng W, Kajigaya S, Chen G, Risitano AM, Nunez O, Young NS (2004). Transcript profile of CD4+ and CD8+ T cells from the bone marrow of acquired aplastic anemia patients. Exp Hematol.

[B11] Szaniszlo P, Wang N, Sinha M, Reece LM, Van Hook JW, Luxon BA, Leary JF (2004). Getting the right cells to the array: Gene expression microarray analysis of cell mixtures and sorted cells. Cytometry.

[B12] Qudes (2005). Prostate cell transcriptome raw data files. http://scgap.systemsbiology.net/data.

[B13] Bogenrieder T, Finstad CL, Freeman RH, Papandreou CN, Scher HI, Albino AP, Reuter VE, Nanus DM (1997). Expression and localization of aminopeptidase A, aminopeptidase N, and dipeptidyl peptidase IV in benign and malignant human prostate tissue. Prostate.

[B14] Kramer G, Steiner G, Fodinger D, Fiebiger E, Rappersberger C, Binder S, Hofbauer J, Marberger M (1995). High expression of a CD38-like molecule in normal prostatic epithelium and its differential loss in benign and malignant disease. J Urol.

[B15] Song J, Aumuller G, Xiao F, Wilhelm B, Albrecht M (2004). Cell specific expression of CD10/neutral endopeptidase 24.11 gene in human prostatic tissue and cells. Prostate.

[B16] Reis-Filho JS, Simpson PT, Martins A, Preto A, Gartner F, Schmitt FC (2003). Distribution of p63, cytokeratins 5/6 and cytokeratin 14 in 51 normal and 400 neoplastic human tissue samples using TARP-4 multi-tumor tissue microarray. Virchows Arch.

[B17] Yang Y, Hao J, Liu X, Dalkin B, Nagle RB (1997). Differential expression of cytokeratin mRNA and protein in normal prostate, prostatic intraepithelial neoplasia, and invasive carcinoma. Am J Pathol.

[B18] Dehan P, Waltregny D, Beschin A, Noel A, Castronovo V, Tryggvason K, De Leval J, Foidart JM (1997). Loss of type IV collagen alpha 5 and alpha 6 chains in human invasive prostate carcinomas. Am J Pathol.

[B19] Liu AY, Roudier MP, True LD (2004). Heterogeneity in primary and metastatic prostate cancer as defined by cell surface CD profile. Am J Pathol.

[B20] Danielpour D (2005). Functions and regulation of transforming growth factor-beta (TGF-beta) in the prostate. Eur J Cancer.

[B21] Hildebrand A, Romaris M, Rasmussen LM, Heinegard D, Twardzik DR, Border WA, Ruoslahti E (1994). Interaction of the small interstitial proteoglycans biglycan, decorin and fibromodulin with transforming growth factor beta. Biochem J.

[B22] Ruzinova MB, Benezra R (2003). Id proteins in development, cell cycle and cancer. Trends Cell Biol.

[B23] Hollnagel A, Oehlmann V, Heymer J, Ruther U, Nordheim A (1999). Id genes are direct targets of bone morphogenetic protein induction in embryonic stem cells. J Biol Chem.

[B24] Lamm ML, Podlasek CA, Barnett DH, Lee J, Clemens JQ, Hebner CM, Bushman W (2001). Mesenchymal factor bone morphogenetic protein 4 restricts ductal budding and branching morphogenesis in the developing prostate. Dev Biol.

[B25] Almahbobi G, Hedwards S, Fricout G, Jeulin D, Bertram JF, Risbridger GP (2005). Computer-based detection of neonatal changes to branching morphogenesis reveals different mechanisms of and predicts prostate enlargement in mice haplo-insufficient for bone morphogenetic protein 4. J Pathol.

[B26] Brubaker KD, Corey E, Brown LG, Vessella RL (2004). Bone morphogenetic protein signaling in prostate cancer cell lines. J Cell Biochem.

[B27] Luo J, Dunn T, Ewing C, Sauvageot J, Chen Y, Trent J, Isaacs W (2002). Gene expression signature of benign prostatic hyperplasia revealed by cDNA microarray analysis. Prostate.

[B28] Rubin MA, Zhou M, Dhanasekaran SM, Varambally S, Barrette TR, Sanda MG, Pienta KJ, Ghosh D, Chinnaiyan AM (2002). alpha-Methylacyl coenzyme A racemase as a tissue biomarker for prostate cancer. Jama.

[B29] Dhanasekaran SM, Barrette TR, Ghosh D, Shah R, Varambally S, Kurachi K, Pienta KJ, Rubin MA, Chinnaiyan AM (2001). Delineation of prognostic biomarkers in prostate cancer. Nature.

[B30] Lin B, White JT, Ferguson C, Wang S, Vessella R, Bumgarner R, True LD, Hood L, Nelson PS (2001). Prostate short-chain dehydrogenase reductase 1 (PSDR1): a new member of the short-chain steroid dehydrogenase/reductase family highly expressed in normal and neoplastic prostate epithelium. Cancer Res.

[B31] Afar DE, Vivanco I, Hubert RS, Kuo J, Chen E, Saffran DC, Raitano AB, Jakobovits A (2001). Catalytic cleavage of the androgen-regulated TMPRSS2 protease results in its secretion by prostate and prostate cancer epithelia. Cancer Res.

[B32] St Croix B, Rago C, Velculescu V, Traverso G, Romans KE, Montgomery E, Lal A, Riggins GJ, Lengauer C, Vogelstein B, Kinzler KW (2000). Genes expressed in human tumor endothelium. Science.

[B33] Gentleman RC, Carey VJ, Bates DM, Bolstad B, Dettling M, Dudoit S, Ellis B, Gautier L, Ge Y, Gentry J, Hornik K, Hothorn T, Huber W, Iacus S, Irizarry R, Leisch F, Li C, Maechler M, Rossini AJ, Sawitzki G, Smith C, Smyth G, Tierney L, Yang JY, Zhang J (2004). Bioconductor: open software development for computational biology and bioinformatics. Genome Biol.

[B34] Kanehisa M, Goto S (2000). KEGG: Kyoto encyclopedia of genes and genomes. Nucleic Acids Res.

