# Supplementary figures and images for: Transcriptomes of human prostate cells
Source: BMC Genomics. 2006 Apr 25;7:92. doi: 10.1186/1471-2164-7-92 (PMC1553448; doi:10.1186/1471-2164-7-92)

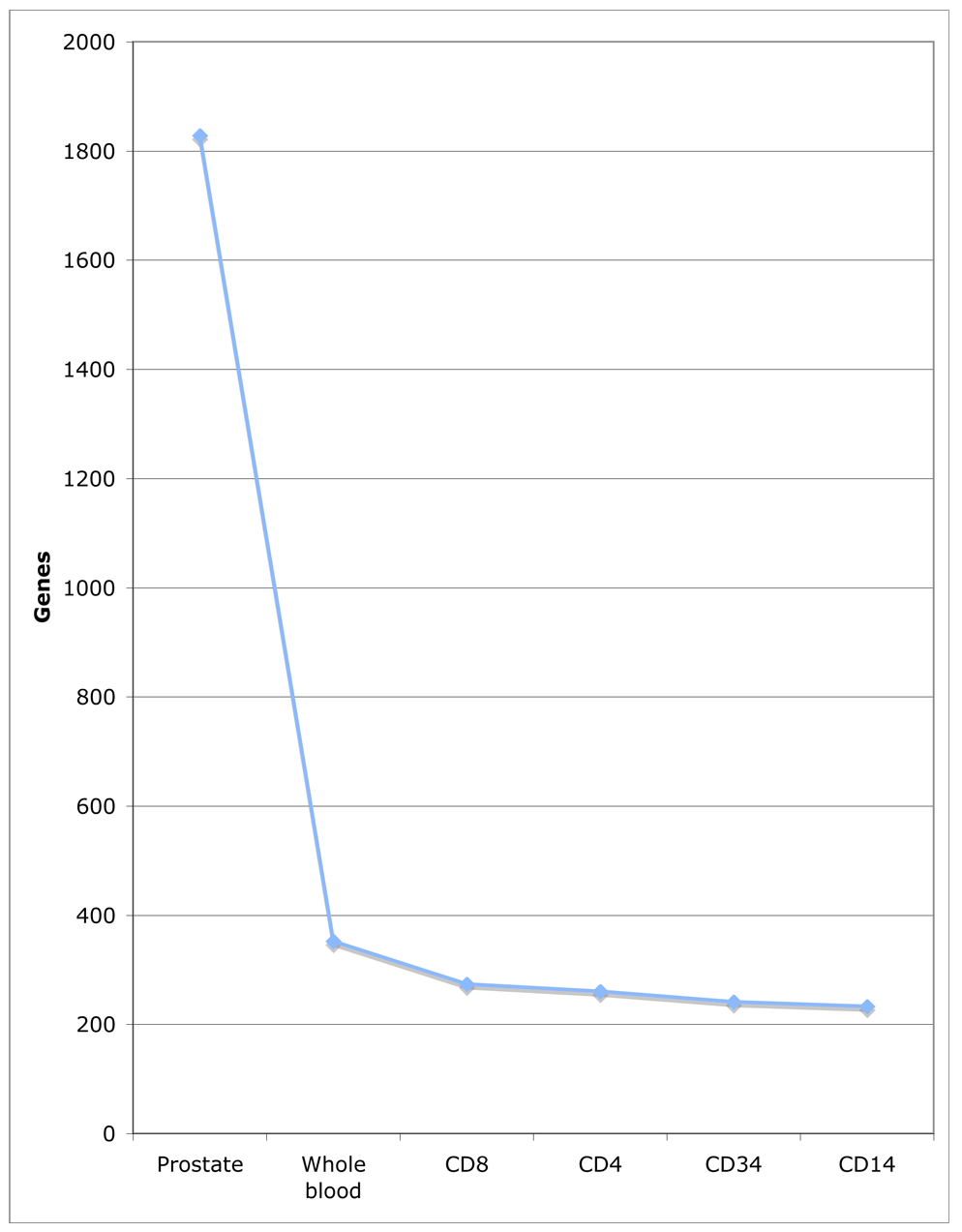

Supplement: Additional file 3 — Subtraction of blood-cell genes from prostate transcriptomes Number of genes (y-axis) detected in whole prostate tissue but not sorted (x-axis point “Prostate”). The remaining x-axis points indicate the blood-cell type which was subtracted in silico from the whole prostate data and the resulting number of genes is plotted on the y-axis. Subtraction of whole blood expressed genes from whole prostate data accounted for 88% of genes detected in whole prostate but not sorted cells. [file 1471-2164-7-92-S3.tiff]
